# Supplementary material for: Colloidal gelation with non-sticky particles
Source: Nat Commun. 2023 May 15;14:2773. doi: 10.1038/s41467-023-38461-1 (PMC10185553; doi:10.1038/s41467-023-38461-1)
Supplement: Supplementary file 1 — Supplementary information [file 41467_2023_38461_MOESM1_ESM.pdf]

Supplementary Information for "Colloidal Gelation with Non-Sticky Particles"

Yujie Jiang[1,2,3,*] and Ryohei Seto[1,3,4,†]

[1] *Wenzhou Key Laboratory of Biomaterials and Engineering, Wenzhou Institute, University of Chinese Academy of Sciences, Wenzhou, Zhejiang 325000, China*

[2] *School of Physical Sciences, University of Chinese Academy of Sciences, Beijing 100049, China*

[3] *Oujiang Laboratory (Zhejiang Lab for Regenerative Medicine, Vision and Brain Health), Wenzhou, Zhejiang 325000, China*

[4] *Graduate School of Information Science, University of Hyogo, Kobe, Hyogo 650-0047, Japan*

(Dated: April 23, 2023)

[*] jiangyujie@ucas.ac.cn

[†] seto@ucas.ac.cn

SUPPLEMENTARY NOTE 1: GEL AND GLASS

As volume fraction ϕ increases, attractive colloids transition from ramified gel into attractive glass (AG) [1]. One signature of AG is structural homogeneity, usually evident by the flat structure factor $S(q)$ at small wavenumber q. Starting from a random homogeneous state, our attractive systems at four ϕ all exhibit dynamical arrest upon $t = 100\tau_{\mathrm{B}}$, suggested by the MSD plateau in Supplementary Fig. 1 **a**. We then measure the structure factor $S(q)$ of each system at steady states ($t = 100\tau_{\mathrm{B}}$), Supplementary Fig. 1 **b**. The absence of low-q peak indicates a homogeneous AG state at $\phi = 0.5$, while the other three concentrations all exhibit heterogeneity at large lengthscale. This explains the deviation of t_{g} from the power-fitting at high ϕ, Fig. 1 **c** in the main text. We therefore identify a AG region with $\phi > 0.4$.

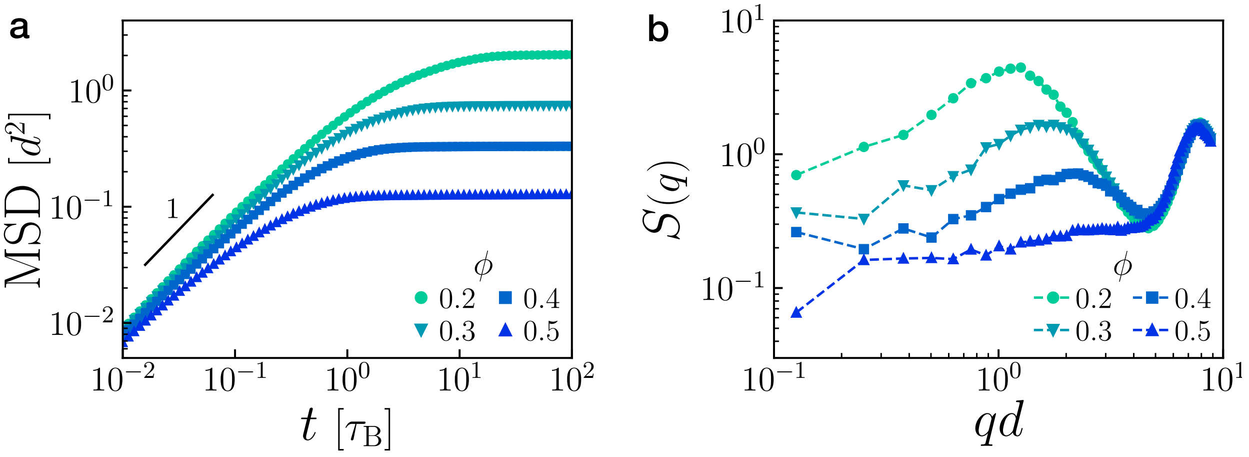

Supplementary Fig. 1. **a** Mean squared displacement (MSD) of attractive colloids at different volume fractions ϕ. All systems start from a random homogeneous configuration at $t = 0$. **b** Static structure factor $S(q)$ of attractive gels at $t = 100\tau_{\mathrm{B}}$.

SUPPLEMENTARY NOTE 2: STRUCTURE FACTOR $S(q)$ AT GELATION

The characteristic lengthscale ξ_g in Fig. 3**a** (main text) is derived from the structure factor $S(q)$ at gelation points. Supplementary Fig. 2 shows $S(q)$ at the gelation points of attractive gels (**a**) and adhesive gels (**b**), respectively. As volume fraction ϕ increases, the peak position q_0 shifts to larger wavenumbers in both cases, suggesting that $\xi_\mathrm{g} \equiv 2\pi/q_0$ decreases with ϕ.

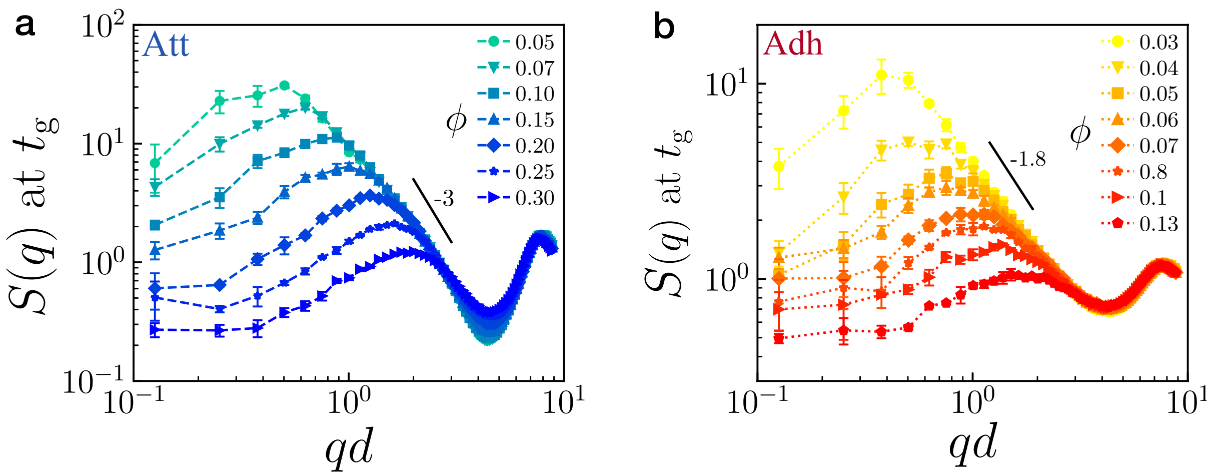

Supplementary Fig. 2. **Structure factor $S(q)$ at gelation points (isostaticity percolation). a** and **b** respectively refer to attractive and adhesive gels of different volume fractions ϕ.

SUPPLEMENTARY NOTE 3: GEL LENGTHSCALE IN COMPOSITE SYSTEMS

In the main text, we assume that the ξ_g–ϕ power-law in colloidal gels also applies to binary composites by substituting $\phi_{\rm eff}$ for ϕ, i.e., $\xi_g = \xi_g(\phi_{\rm eff})$. We did not extract ξ_g directly from the colloid–colloid $S(q)$ of binary systems, because in most cases, the presence of large NS particles distorts the structure at large scale (low q) and dominates over the lengthscale in gel matrix, such as Supplementary Fig. 3 **a**. Peak of ξ_g becomes resolvable at smaller $d_{\rm NS}$ (Supplementary Fig. 3 **b**), where systems with the same $\phi_g = 0.1$ yet different $\phi_{\rm NS}$ present an identical $S(q)$ curve at gelation points. Despite of different gelation times t_g as shown in Supplementary Fig. 5 **a**, these systems have similar structures (excluding NS particles) with the pure gel. Thus, the real effective concentration reduces to ϕ_g.

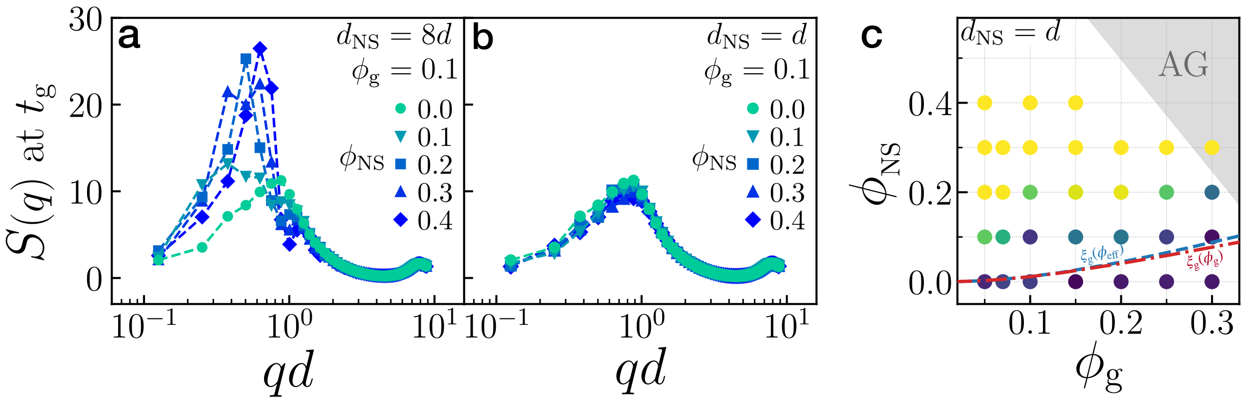

Supplementary Fig. 3. **Colloid-colloid structure factor $S(q)$ at the gelation points of attractive systems at $\phi_g = 0.1$.** System with $\phi_{\rm NS} = 0$ refers to colloidal gel and those with $\phi_{\rm NS} > 0$ refer to binary composites. **a** $d_{\rm NS} = 8d$. **b** $d_{\rm NS} = d$. **c** Diagram of attractive systems at $d_{\rm NS} = d$ with contour lines $\gamma = 2$ calculated from $\xi_g(\phi_{\rm eff})$ (blue) and $\xi_g(\phi_g)$ (red).

To better characterize the gel length in composites with large NS, we use an alternative method adopted from [2]. We first divide the space into cubic grids with the mesh much smaller than particles ($d_{\rm mesh} = 0.1d$). For each lattice point, we define the void volume $V_{\rm void}$ as the volume of a sphere in contact with the nearest-neighbor particle, with the center of the sphere at the lattice point, Supplementary Fig. 4 **a**. A void lengthscale $\xi_{\rm void} = \sqrt[3]{V_{\rm void}}$ can then be counted. For pure gels, the distribution of $\xi_{\rm void}$ varies with the volume fraction ϕ, and the peak position (corresponding to a characteristic void length) shifts to lower as

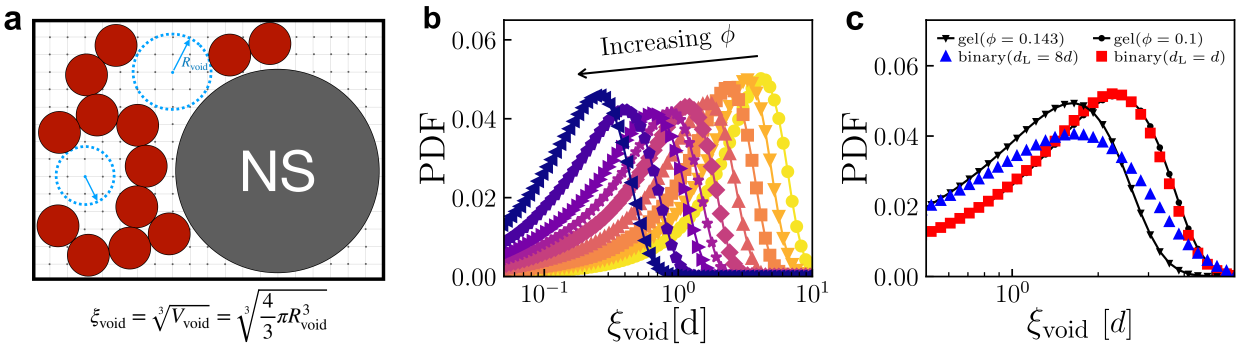

Supplementary Fig. 4. **Void length distribution.** **a** Schematic method to determine void distribution. **b** Probability density function (PDF) of void length $\xi_{\rm void}$ in attractive gels. **c** Distribution of $\xi_{\rm void}$ in colloidal gels (black) and binary composites with $\phi_{\rm g} = 0.1$ and $\phi_{\rm NS} = 0.3$ (so that $\phi_{\rm eff} = 0.143$).

ϕ increases, Supplementary Fig. 4 **b**. This is consistent with $\xi_{\rm g}$ decreasing with ϕ in Fig. 3 **a** (main text).

For binary composites, we exclude the lattice points inside NS particles so that only the voids within gel matrix are counted. Here we compare composites with $\phi_{\rm g} = 0.1$ and $\phi_{\rm NS} = 0.3$ (so that $\phi_{\rm eff} = 0.143$), and gels with $\phi = 0.1$ and $\phi = 0.143$, respectively. At small $d_{\rm NS} = d$, the $\xi_{\rm void}$ distribution of binary composite is almost identical to that of $\phi = \phi_{\rm g} = 0.1$ gel (Supplementary Fig. 4 **c**), consistent with the collapsed $S(q)$ in Supplementary Fig. 3 **b**. At large $d_{\rm NS} = 8d$, by contrast, the peak position of composite coincides with a gel at $\phi = \phi_{\rm eff} = 0.143$, Supplementary Fig. 4 **c**. This suggests that the gel length in a mixture is comparable to that in a pure gel system with $\phi = \phi_{\rm eff}$. The small difference in the distribution may stem from the spherical boundary of NS particles.

These results justify our assumption, i.e., *using the gel length for an equivalent pure system as a proxy for the the gel length in the mixture* is reasonable. Though there is a variation from $\phi_{\rm eff}$ to $\phi_{\rm g}$ for different NS size, we have shown that this has little effect on the iso-γ curve, Supplementary Fig. 3 **c**.

SUPPLEMENTARY NOTE 4: DISTRIBUTIONS OF THE LENGTHSCALES

The two lengthscales in the ratio γ both have distributions. In a system of only NS particles, the probability density function of δ varies with the NS concentration $\phi_{\rm NS}$ as shown in Supplementary Fig. 5 **a**. As $\phi_{\rm NS}$ decreases, both the width of peak and the mean value decreases. During gelation, the lengthscale δ barely varies over time regardless of other parameters (inset). This suggests the time-independence of δ.

In the main text, we consider $\xi_{\rm g}$ as a characteristic size of gel clusters. Another quantity to represent cluster size is the radius of gyration $R_{\rm g}$. In Supplementary Fig. 5 **b**, we show the distribution of $2R_{\rm g}$ at different stages during gelation of $\phi = 0.1$ attractive system. As gelation proceeds, clusters aggregate into a large, primary cluster so that the number of small agglomerates decreases over time.

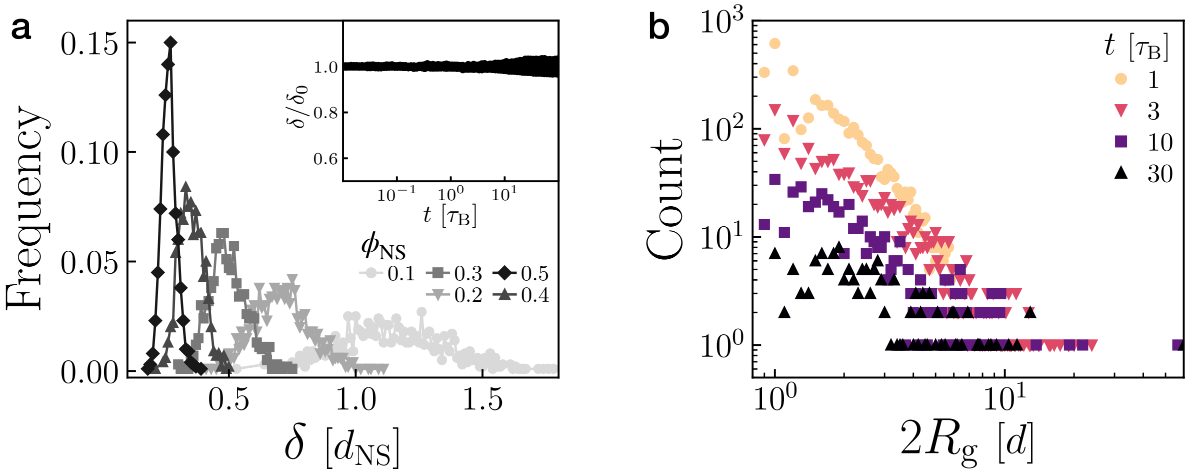

Supplementary Fig. 5. **Distributions of lengthscales.** **a** Distribution of NS particle interstice δ at different volume fractions $\phi_{\rm NS}$. Inset: variation of normalized δ/δ_0 (with $\delta_0 \equiv \delta(t = 0)$) in composite systems with various compositions and NS particle sizes. **b** Distribution of cluster size $2R_g$ (R_g refers to gyration radius) in a colloidal gel ($\phi = 0.1$) at different stages.

SUPPLEMENTARY NOTE 5: RAW DATA OF DIAGRAMS

Supplementary Fig. 6 shows all the gelation time t_g data required for the diagrams in Fig. 3 **d** and **e** (main text).

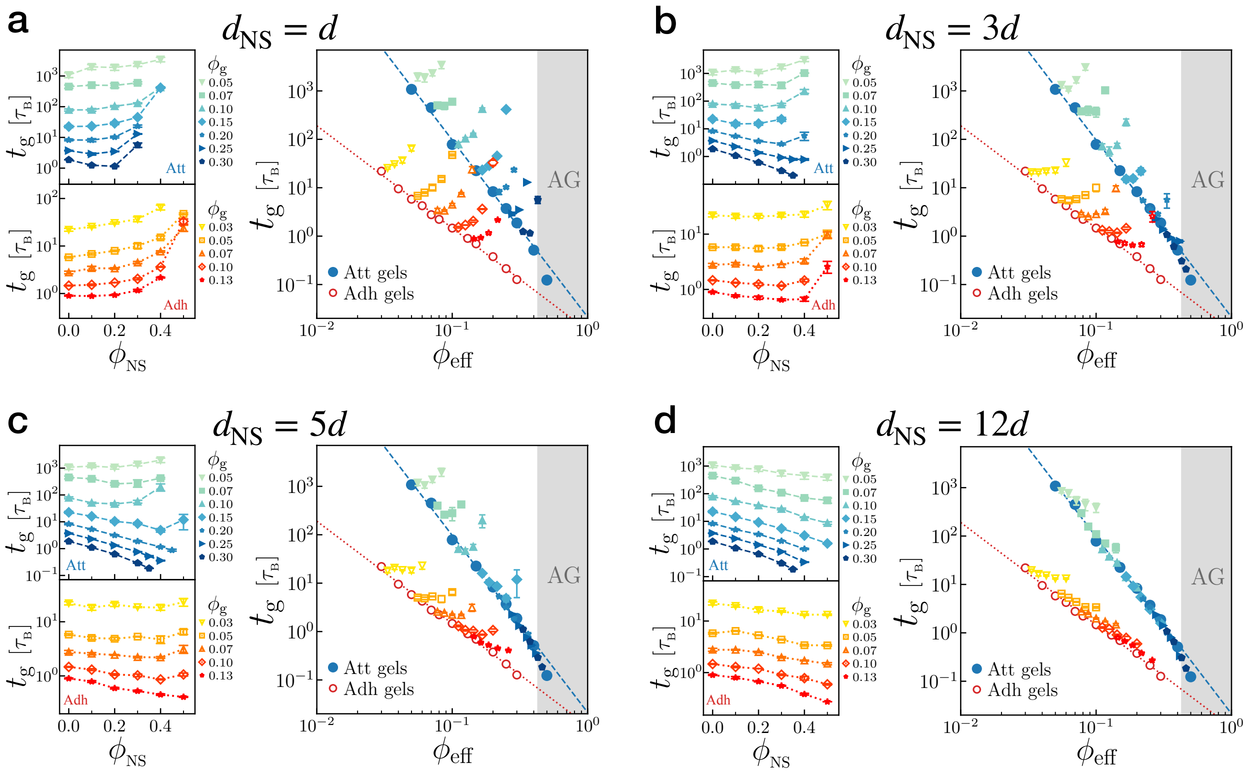

Supplementary Fig. 6. **Gelation time t_g as functions of ϕ_{NS} (left) and ϕ_{eff} (right) at four NS particle sizes.** **a** $d_{NS} = d$, **b** $d_{NS} = 3d$, **c** $d_{NS} = 5d$, and **d** $d_{NS} = 12d$.

SUPPLEMENTARY NOTE 6: CLUSTER MORPHOLOGY

While NS particles affect gelation dynamics, it seems that they play minor role in the shape of gel clusters. To quantify the cluster morphology, we use a dimensionless asphericity (adapted from [3–5]) defined as:

$$\text{Normalized Asphericity (NA)} = \frac{\lambda_k^2 - \frac{1}{2}\left(\lambda_i^2 + \lambda_j^2\right)}{R_\text{g}^2}, \tag{1}$$

where $R_\text{g} = \sqrt{\lambda_i^2 + \lambda_j^2 + \lambda_k^2}$ refers to the gyration radius and $\lambda_{i,j,k}$ to the eigenvalues (ascending order) of the gyration tensor. In particular, NA$= 0$ represents spherically symmetric morphology while NA$= 1$ represents a linear chain of particles. We compare its distribution in gels ($\phi = 0.07$) and binary composites ($\phi_\text{g} = 0.07$ and $\phi_\text{NS} = 0.3$) at different NS sizes, Supplementary Fig. 7. For each sample, we pick the snapshot at $t \approx t_\text{g}/2$ and count for over 1000 clusters consisting more than 10 particles. For mixtures either at the critical boundary ($d_\text{NS} = 8d \to \gamma \approx 2$) or beyond it ($d_\text{NS} = 3d \to \gamma \approx 5.3$), we do not observe significant difference in the asphericity.

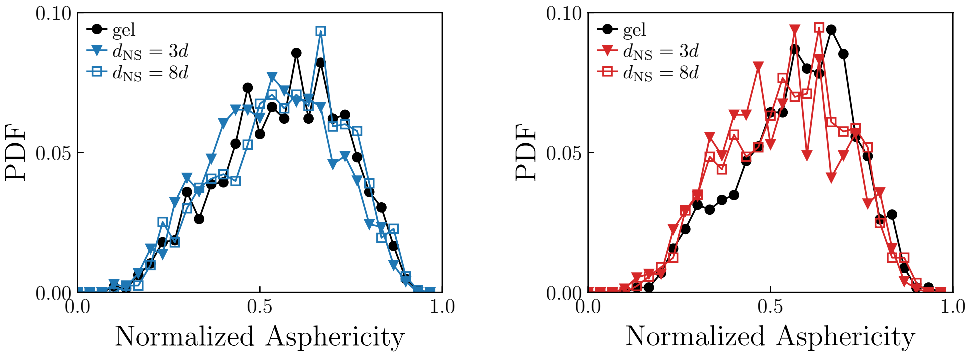

Supplementary Fig. 7. **Asphericity of gel clusters before gelation.** For different contact models (Att – left; Adh – right), we compare a gel at $\phi = 0.07$ and two composites of $\phi_\text{g} = 0.07$ and $\phi_\text{NS} = 0.3$, with different NS sizes corresponding to $\gamma \approx 2$ ($d_\text{NS} = 8d$) and $\gamma \approx 5.3$ ($d_\text{NS} = 3d$), respectively. For each sample, we pick the frame at around $t \approx t_\text{g}/2$ and analysis over 1000 clusters composed of more than 10 colloids.

SUPPLEMENTARY NOTE 7: BACKGROUND VISCOSITY η_{NS}

As mentioned in the Discussion section, the gelation time of pure gels and binary composites (at small d_{NS} limit) can be respectively expressed as:

$$\begin{aligned} t_{\mathrm{g}}^{\mathrm{gel}} &\sim \eta_{\mathrm{f}} \phi^{\alpha}, \\ t_{\mathrm{g}} &\sim \eta_{\mathrm{NS}} \left(\phi_{\mathrm{g}}\right)^{\alpha}. \end{aligned} \tag{2}$$

In this way, the relative background viscosity $\eta_{\mathrm{r}} \equiv \eta_{\mathrm{NS}}/\eta_{\mathrm{f}}$ can be written in a normalized gelation time:

$$\eta_{\mathrm{r}} = \frac{t_{\mathrm{g}}}{t_{\mathrm{g}}^{\mathrm{gel}}} \tag{3}$$

with $\phi = \phi_{\mathrm{g}}$. For all composites with small $d_{\mathrm{NS}} = d$, we plot η_{r} versus ϕ_{NS} in Supplementary Fig. 8. Despite various ϕ_{g}, we find that these data more or less collapse and roughly agree with suspension viscosity in the Krieger–Dougherty form $(1-\phi/\phi_m)^{-2.5\phi_m}$ [6] with $\phi_m = 0.58$ (solid line). Note that the above argument does not consider the volume occupied by the colloids, as it is challenging to quantitatively exclude the gel part. Moreover, our simulations do not take hydrodynamics (e.g., stresslet and pair interaction) into account, so moderate deviation from theory is acceptable.

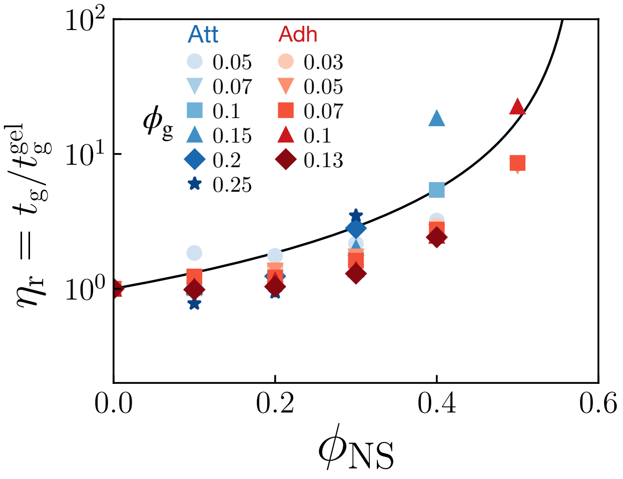

Supplementary Fig. 8. **Relative background viscosity as a function of NS concentration.** Data are from composite systems with small NS ($d_{\mathrm{NS}} = d$). Solid line is the Krieger–Dougherty form $(1-\phi/\phi_m)^{-2.5\phi_m}$ [6], where we use $\phi_m = 0.58$.

SUPPLEMENTARY NOTE 8: PINNED NS PARTICLES

To better understand the role of NS particles, we pin the NS particles ($d_{\mathrm{NS}} = 5d$) during gelation so that they form a fixed porous medium for attractive colloids. We find that the gelation time t_{g} is barely affected below $\gamma = 2$, where the confinement effect dominates. System with $\gamma > 2$, by contrast, do not form percolation even upon $t = 10^4\tau_{\mathrm{B}}$ (open symbols in Supplementary Fig. 9 **c**). This is because rearrangement of NS particles is required for gelation beyond $\gamma = 2$. In such cases, pinning NS particles effectively leads to an infinite background viscosity $\eta_{\mathrm{eff}} \to \infty$.

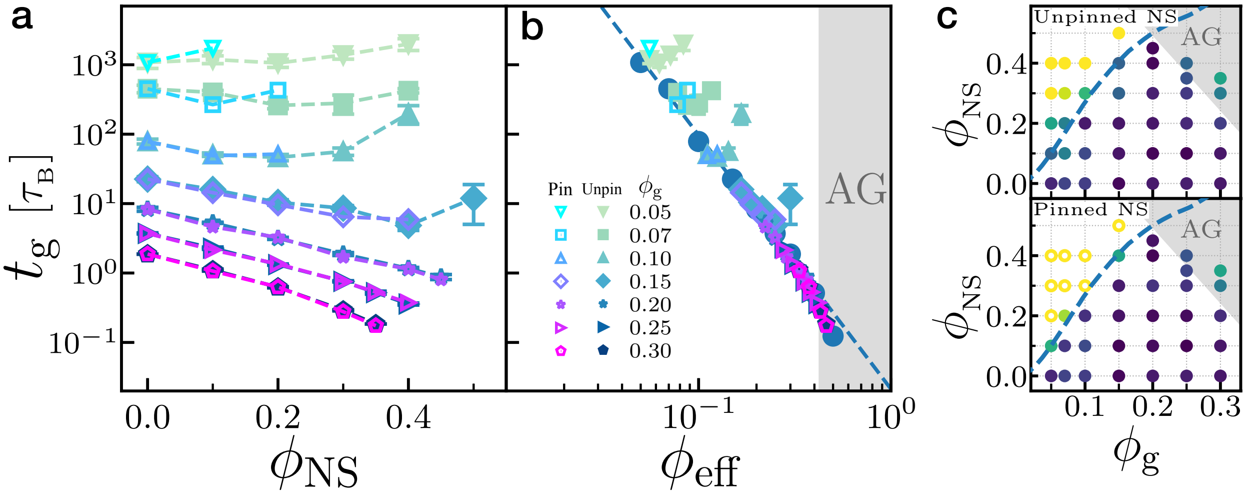

Supplementary Fig. 9. **Comparison between systems with pinned (open) and unpinned (filled) NS particles at $d_{\mathrm{NS}} = 5d$.** **a** and **b** are gelation time t_{g} as functions of ϕ_{NS} and ϕ_{eff}, respectively. **c** ϕ_{g}–ϕ_{NS} diagrams with unpinned NS particles (upper, taken from Figure 3d in the main text) and pinned NS particles (lower). Open symbols represent diverging t_{g}, i.e., samples do not gel within $10^4\tau_{\mathrm{B}}$.

SUPPLEMENTARY NOTE 9: SIMULATION DETAILS

Parameter Setting

We use Langevin dynamics to implement Brownian motion. For pure gel simulations, here we consider four timescales in ascending order: time step dt, damping time $t_{\mathrm{damp}} \equiv \frac{m}{3\pi\eta d}$, interaction time unit $t_{\mathrm{inter}} \equiv \sqrt{\frac{md^2}{U_{\mathrm{att}}}}$ [7] and Brownian time τ_{B}. We expect $dt \ll t_{\mathrm{damp}} \ll t_{\mathrm{inter}} \ll \tau_{\mathrm{B}}$ with reasons listed below:

$$
\begin{aligned}
dt \ll t_{\mathrm{damp}} &: \text{stable simulation} \\
t_{\mathrm{damp}} \ll t_{\mathrm{inter}} &: \text{overdamped condition} \\
t_{\mathrm{damp}} \ll \tau_{\mathrm{B}} &: \text{diffusivity within gelation timescale} \\
t_{\mathrm{inter}} \ll \tau_{\mathrm{B}} &: \text{stiff particles.}
\end{aligned}
$$

In particular, we set $dt = 2 \times 10^{-2} t_{\mathrm{damp}} = 1.2 \times 10^{-3} t_{\mathrm{inter}} = 2 \times 10^{-5} \tau_{\mathrm{B}}$, where timescales are well separated and the computing cost ($\propto \tau_{\mathrm{B}}/dt$) remains appropriate.

The case in binary composite is more complicated, as we simulate with large size ratio of NS to colloids ($d_{\mathrm{NS}}/d = 1$ to 12) and the size difference is expected to be reflected in different mass. If simply applying constant mass density (i.e., $m_i \propto {d_i}^3$), the range of t_{damp} and t_{inter} will be broadened so that the separation of timescales requires higher τ_{B} (i.e., higher computing cost). Under this context, we apply $m_i \propto d_i$ to set a constant t_{damp} for all particles and $t_{\mathrm{damp}} = 10^{-3}\tau_{\mathrm{B}} \ll \tau_{\mathrm{B}}$ to ensure that both colloids and NS particles are diffusive within the relevant time range for gelation process ($\gtrsim 0.1\tau_{\mathrm{B}}$).

Independence

The independence discussed below confirms the robustness of our results.

System Size To verify the independence on system size, we simulate three individual systems (one gel and two composites) at three box sizes ($L_{\mathrm{box}}/d = 35$, 50, and 70). The result of each system is shown in Supplementary Fig. 10, in which we hardly see the system-size dependence around the system size we investigated ($L_{\mathrm{box}}/d = 50$).

Pre-relaxing Time We vary the pre-relaxing time t_{relax} over a wide range before switching on the colloidal attraction to gelation. For both NS spacing δ and gelation time t_{g}, we do not observe systematic dependence on t_{relax}, Supplementary Fig. 11.

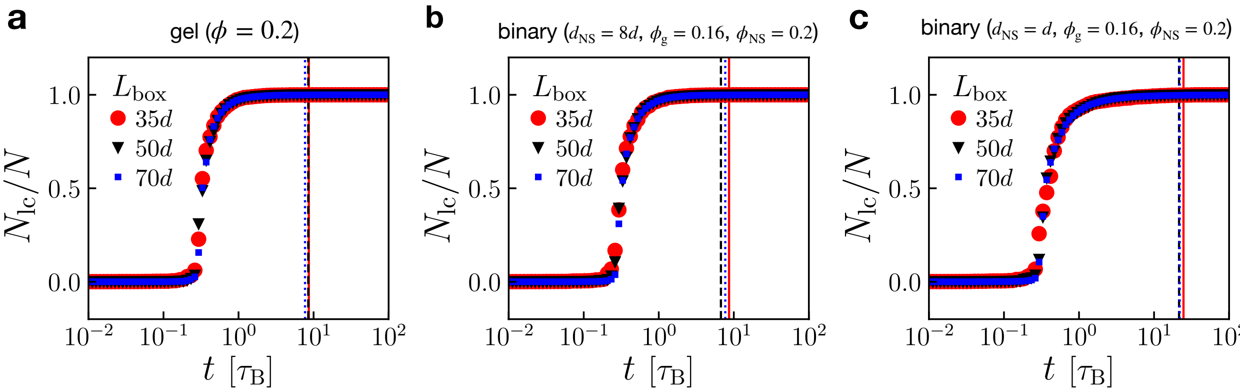

Supplementary Fig. 10. **System size independence.** We measure the largest cluster growth in three different systems (see detailed compositions on the top of each graph) with three different box sizes. Lines represent gelation point $t_{\rm g}$.

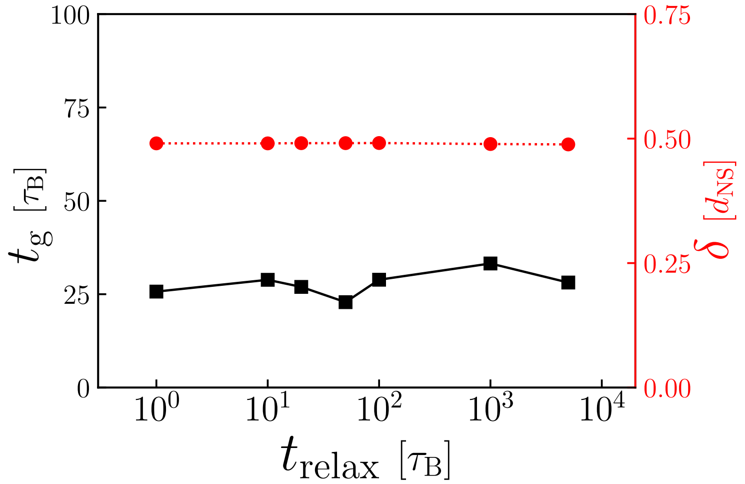

Supplementary Fig. 11. **Independence of pre-relaxing time $t_{\rm relax}$.** We use an attractive system of $\phi_{\rm g} = 0.1$, $\phi_{\rm NS} = 0.3$ and $d_{\rm NS} = 8d$, and vary $t_{\rm relax}$ before switching on the colloidal attraction.

[1] Zaccarelli, E. & Poon, W. C. Colloidal glasses and gels: The interplay of bonding and caging. *Proceedings of the National Academy of Sciences* **106**, 15203–15208 (2009).

[2] Koumakis, N. *et al.* Tuning colloidal gels by shear. *Soft Matter* **11**, 4640–4648 (2015).

[3] Sanchez, R. & Bartlett, P. Equilibrium cluster formation and gelation. *J. Condens. Matter Phys.* **17**, S3551 (2005).

[4] Whitmer, J. K. & Luijten, E. Influence of hydrodynamics on cluster formation in colloid-polymer mixtures. *J. Phys. Chem. B* **115**, 7294–7300 (2011).

[5] Fry, D., Mohammad, A., Chakrabarti, A. & Sorensen, C. M. Cluster shape anisotropy in irreversibly aggregating particulate systems. *Langmuir* **20**, 7871–7879 (2004).

[6] Krieger, I. M. & Dougherty, T. J. A mechanism for non-newtonian flow in suspensions of rigid spheres. *Trans. Soc. Rheol.* **3**, 137–152 (1959).

[7] Ferreiro-Córdova, C. *et al.* Stiffening colloidal gels by solid inclusions. *Soft Matter* **18**, 2842–2850 (2022).
